# Cytokine-based Predictive Models to Estimate the Probability of Chronic Periodontitis: Development of Diagnostic Nomograms

**DOI:** 10.1038/s41598-017-06674-2

**Published:** 2017-09-14

**Authors:** I. Tomás, N. Arias-Bujanda, M. Alonso-Sampedro, M. A. Casares-de-Cal, C. Sánchez-Sellero, D. Suárez-Quintanilla, C. Balsa-Castro

**Affiliations:** 1 Oral Sciences Research Group, Special Needs Unit, Department of Surgery and Medical-surgical Specialties, School of Medicine and Dentistry, Health Research Institute of Santiago (IDIS), Universidade de Santiago de Compostela, Galicia, Spain; 2 Department of Internal Medicine and Clinical Epidemiology, Complejo Hospitalario Universitario, Santiago de Compostela, Galicia, Spain; 3 Department of Mathematical Analysis, Statistics and Optimization, School of Mathematics, Universidade de Santiago de Compostela, Galicia, Spain

## Abstract

Although a distinct cytokine profile has been described in the gingival crevicular fluid (GCF) of patients with chronic periodontitis, there is no evidence of GCF cytokine-based predictive models being used to diagnose the disease. Our objectives were: to obtain GCF cytokine-based predictive models; and develop nomograms derived from them. A sample of 150 participants was recruited: 75 periodontally healthy controls and 75 subjects affected by chronic periodontitis. Sixteen mediators were measured in GCF using the Luminex 100™ instrument: GMCSF, IFNgamma, IL1alpha, IL1beta, IL2, IL3, IL4, IL5, IL6, IL10, IL12p40, IL12p70, IL13, IL17A, IL17F and TNFalpha. Cytokine-based models were obtained using multivariate binary logistic regression. Models were selected for their ability to predict chronic periodontitis, considering the different role of the cytokines involved in the inflammatory process. The outstanding predictive accuracy of the resulting smoking-adjusted models showed that IL1alpha, IL1beta and IL17A in GCF are very good biomarkers for distinguishing patients with chronic periodontitis from periodontally healthy individuals. The predictive ability of these pro-inflammatory cytokines was increased by incorporating IFN gamma and IL10. The nomograms revealed the amount of periodontitis-associated imbalances between these cytokines with pro-inflammatory and anti-inflammatory effects in terms of a particular probability of having chronic periodontitis.

## Introduction

Periodontal diseases are among the most common conditions affecting humans^1^. In 2010, severe periodontitis was estimated to be the sixth most prevalent disease globally, affecting 743 million people worldwide and with an age-standardized incidence of 701 cases per 100,000 person-years^2^.

A recent prevalence study of adult periodontitis in the USA, with data from the 2009 and 2010 National Health and Nutrition Examination Survey, revealed that over 47% of individuals had periodontitis, which equates to 64.7 million adults^3, 4^. During the recent 11th European Workshop on Periodontology, experts confirmed that the prevalence of periodontitis in Europe remains high, affecting more than 50% of the adult population and, in its severe forms, 11% of adults^5^. Periodontitis is not a “silent” problem: periodontal patients have a poorer perception of their oral health and a worse quality of life compared to healthy individuals, with periodontal treatment improving the oral health-related quality of life of these people^6, 7^.

On the other hand, periodontitis is currently being connected bidirectionally to the pathogenesis of various systemic diseases and conditions such as diabetes^8^; coronary heart disease^9^; rheumatoid arthritis^10^; respiratory diseases^11^; pre-term low birth weights^12^; and dementia^13^.

Traditional clinical measures are informative for evaluating the severity of periodontitis and the response to therapy, and these include: the presence or the level of bacterial plaque; gingival inflammation and bleeding upon probing; pocket depth and suppuration; the clinical attachment level; and radiographic bone loss^14^. Nevertheless, these clinical criteria are unable to determine current disease activity or the future risk of structure loss^15, 16^. As a result, one of the major challenges in the field of Periodontology is to determine biomarkers for screening and predicting the early onset of periodontitis or evaluating the disease activity as well as the efficacy of therapy (diagnostic or prognostic tests)^15, 17^.

The primary hallmark of periodontitis, namely the destruction of periodontal tissue, is widely accepted to be a result of a chronic inflammatory host immune response caused by a polymicrobial dysbiosis^18, 19^. This host immune response is characterized by: 1) infiltration of the gingival tissues by neutrophils, monocytes/macrophages and lymphocytes; and 2) the generation of high concentrations of mediators, including cytokines, chemokines, arachidonic acid metabolites and proteolytic enzymes^18, 20^. The nature and extent of this host immune response are fundamental determinants of the susceptibility to and progression of periodontitis^21^.

Gingival crevicular fluid (GCF) is an exudate of the serum originating from the gingival plexus of blood vessels in the gingival connective tissue, close to the epithelium lining of the dentogingival space. With an increase in the severity of periodontal inflammation, the amount of GCF increases significantly. Additionally, its consistency transforms into an inflammatory exudate as it traverses the inflamed tissues, collecting bacterial and host molecules^22, 23^. The collection of GCF is relatively non-invasive, and this sampling method has been shown to capture inflammatory and connective tissue breakdown mediators accurately^23^.

With the establishment of enzyme-linked immunosorbent assay (ELISA) techniques, interleukin (IL) 1beta was the first cytokine to be specifically measured in the gingival tissue of patients with chronic periodontitis^24^. Numerous papers since then have reported the measurement of cytokines in GCF, confirming that there is a distinct cytokine profile for patients with chronic periodontitis^17, 25^. The results concerning which cytokines are most involved in chronic periodontitis are, however, generally contradictory, due to the lack of uniformity in the methodological design of the studies^25^.

On the other hand, very few authors have examined the simultaneous presence of more than 10 cytokines in GCF, and there is a failure to analyse a broader spectrum of biomarkers that may directly influence the local inflammatory response in periodontitis^26–28^. Moreover, there is no evidence in the literature of the development, validation, or updating of GCF cytokine-based predictive models for diagnosing periodontitis or its prognosis using appropriate multivariate predictive modeling techniques^29^. As a consequence, it is quite clear that highly specific and sensitive biomarkers for monitoring periodontitis are still needed for the early and better detection of periodontal tissue destruction^17^.

Accordingly, the objectives of the present cross-sectional study were to:

1. Compare the levels of 16 cytokines detected in GCF between periodontally healthy individuals and patients with chronic periodontitis.
2. Obtain GCF cytokine-based predictive models that could be used to distinguish periodontal patients from periodontally healthy individuals.
3. Develop nomograms derived from GCF cytokine-based predictive models.

## Material and Methods

### Selection of study groups

**Figure 1:**
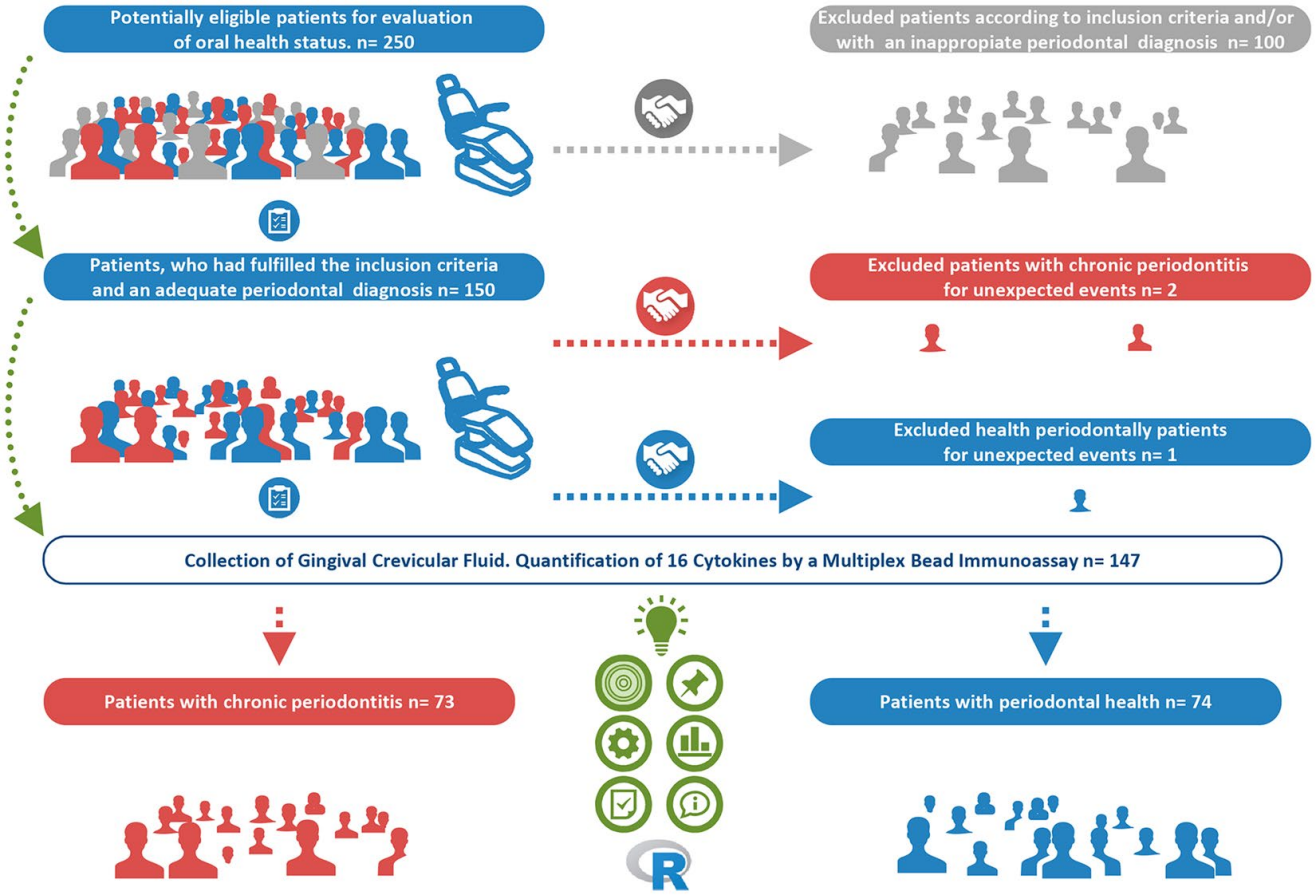
Selection of study groups.

A convenience sample of 150 eligible participants, comprising 75 periodontally healthy controls (control group) and 75 subjects affected by moderate to severe generalized chronic periodontitis (perio group), were recruited among 250 consecutive patients of the general population who were referred to the School of Medicine and Dentistry (Universidade de Santiago de Compostela, Spain) for an assessment of their oral health status between 2013–2015. Patients were selected if they fulfilled the following inclusion criteria: 1) age 30 to 75; 2) no medical history of diabetes mellitus or hepatic or renal disease, or other serious medical conditions or transmittable diseases; 3) no history of alcohol or drug abuse; 4) no pregnancy or breastfeeding; 5) no intake of systemic antimicrobials during the previous six months; 6) no intake of anti-inflammatory medication in the previous four months; 7) no routine use of oral antiseptics; 8) no presence of implants or orthodontic appliances; 9) no previous periodontal treatment; 10) smokers, who had stopped smoking less than five years before the sampling; and 11) the presence of at least 18 natural teeth (Fig. 1).

One experienced dentist, who previously underwent a calibration exercise of the clinical parameters in a small group of patients, performed all the periodontal diagnoses. The probing pocket depth (PPD) and clinical attachment level (CAL) were recorded throughout the mouth on six sites per tooth using a PCP-UNC 15 probe. Bleeding on probing (BOP) and bacterial plaque level (BPL) were recorded for the full mouth on a binary scale (presence/absence) on six sites per tooth (BOP and BPL, respectively). Standardized radiographs of all teeth were obtained to assess the alveolar bone status.

The presence or absence of chronic periodontitis was based on clinical/radiographic information. The control group included periodontally healthy individuals with BOP < 25%, no sites with a PPD ≥ 4 mm and no radiographic evidence of alveolar bone loss. The perio group patients were diagnosed as suffering from moderate to severe generalized chronic periodontitis based on the previously established criteria^30, 31^. Smoking histories were obtained using a questionnaire, with information collected on smoking status (never, past or current), the years (months) of smoking and the number of cigarettes/day. All answers were reviewed with the subject by a member of the study team.

This study was conducted according to the principles outlined in the Declaration of Helsinki (as revised in 2000) on experimentation involving humans^32^. The transparent reporting of a multivariable prediction model for individual prognosis or diagnosis (TRIPOD) guidelines were considered^29^.

### Gingival crevicular fluid sampling

The GCF collection took place one week after the initial examination, and the samples were obtained at the same time of day (in the afternoon, approximately 5–7 h after toothbrushing). Prior to the sampling, the individual tooth site was isolated with cotton rolls, supragingival plaque was carefully removed and the site was gently air-dried with an air syringe. A paper strip (Periopaper, Amityville, NY, USA) was inserted into the gingival sulcus or periodontal pocket for 30 sec. In cases of visible contamination with blood, the strips were discarded and new sites sampled.

GCF samples from the periodontally healthy patients were collected and pooled from 20 sites from teeth in quadrants 1 and 3 (incisor, canine, first premolar, second premolar and molar). In the periodontal patients, subgingival samples were collected and pooled from the deepest PPD sites in each quadrant (a total of 20 non-adjacent proximal sites). Strips from each subject were placed into labelled tubes containing: 300 ml of 0.01M PBS with a pH of 7.2; and a protease inhibitor (Complete Mini, protease inhibitor cocktail tablets, Roche Applied Science, Indianapolis, IN, USA). To prevent evaporative losses, the GCF volume was determined based on immediate measurements of weighing the tubes and strips before and after the sample collection using a very sensitive scale^22^.

After shaking for 20 min, the strips were removed and the eluates centrifuged for 5 min at 5800 g to remove plaque and cellular elements. The GCF samples were then frozen at −80 °C until further biochemical analysis.

### Quantification of cytokines in gingival crevicular fluid using multiplexed bead immunoassays

A single investigator blinded to the clinical data performed the quantitative cytokine analyses. Cytokine levels in the GCF were determined using the human cytokine 16-plex Procarta immunoassay (Affymetrix, Inc., Santa Clara, CA, USA). Sixteen mediators were measured: 1) eight pro-inflammatory cytokines (granulocyte-macrophage colony stimulating factor – GMCSF -, IL1alpha, IL1beta, IL6, IL12p40, IL17A, IL17F and TNFalpha); and 2) eight anti-inflammatory cytokines (IFNgamma, IL2, IL3, IL4, IL5, IL10, IL12p70 and IL13).

The assays were performed in 96-well filter plates following the manufacturer’s instructions. Briefly, the filter plates were pre-wet with washing buffer and the solution was aspirated from the wells using a handheld magnetic separator block (Millipore Corporation, Billerica, MA). Microsphere beads coated with monoclonal antibodies to the 16 different target analytes were added to the wells. Standards and samples were pipetted into the wells and incubated overnight at 4 °C. The wells were then washed, again using a handheld magnetic separator block (Millipore Corporation), and a mixture of biotinylated secondary antibodies was added.

After incubation for 30 min, streptavidin conjugated to the fluorescent protein R-phycoerythrin (streptavidin-RPE) was added to the beads and incubated for 30 min. After washing to remove the unbound reagents, a reading buffer (Affymetrix, Inc) was added to the wells and the beads (minimum of 100 per analyte) were analysed using the Luminex 100™ instrument (Luminex Corporation, Austin, Texas, USA). All the samples were run in duplicate.

The Luminex 100™ monitored the spectral properties of the beads to distinguish the different analytes while simultaneously measuring the amount of fluorescence associated with R-phycoerythrin, which is reported as the median fluorescence intensity (MFI). The concentrations of the unknown samples (antigens in the GCF samples) were: 1) estimated from the standard curve using a 5PL algorithm and the Luminex IS 2.3 and xPONENT 3.1 softwares (Luminex Software, Inc.); and 2) expressed as pg/ml adjusting for the dilution factor. The concentration ranges for each biomarker analysed were: GMCSF, 0.53–55,050 pg/ml; IFNgamma, 0.02–6,650 pg/ml; IL1alpha, 0.34–28,800 pg/ml; IL1beta, 0.09–23,150 pg/ml; IL2, 0.04–13,700 pg/ml; IL3, 0.19–26,500 pg/ml; IL4, 0.10–29,250 pg/ml; IL5, 0.04–17,800 pg/ml; IL6, 0.10–27,200 pg/ml; IL10, 0.04–10,050 pg/ml; IL12p40, 0.14–27,350 pg/ml; IL12p70, 0.26–18,050 pg/ml; IL13, 0.34–23,700 pg/ml; IL17A, 0.36–30,900 pg/ml; IL17F, 0.25–34,700 pg/ml; and TNFalpha, 0.21–16,800 pg/ml.

Samples below the detection limit (DL) of the assay were recorded as DL/2^33^, while samples above the upper limit of quantification of the standard curves were assigned the highest value of the curve.

### Statistical analysis

The statistical analyses were performed using the R software^34^. A supplementary file shows the data frame used in the present study for comparative and predictive analysis (Supplementary Dataset 1).

#### Comparison of clinical characteristics between control individuals and patients with chronic periodontitis

Univariate tests to detect differences in the clinical characteristics between the control and perio groups were performed. The normality assumption of the quantitative variables was checked by the Shapiro-Wilk test, with the result being that the normality hypothesis was not valid in all the cases. Consequently, the Mann-Whitney U test was used to compare the quantitative clinical characteristics between both groups (age, number of teeth and the clinical parameters BPL, BOP and CAL in the full mouth and the sampled sites). The Fisher’s exact test was used to assess the association between the qualitative variables (gender and smoking habit) between both study groups. The significance level applied was *p* < 0.05.

#### Comparison of GCF cytokine levels between control individuals and patients with chronic periodontitis

After applying the Shapiro-Wilk test, and because the distributions of the cytokines were skewed, we used logarithmically transformed values (log_2_) for the statistical analyses. Histograms were made for each variable before and after the logarithmic transformation, showing how the transformation produces a less skewed and more suitable distribution for further analysis.

Quantitative data on the GCF cytokine levels were expressed as medians and interquartile ranges. The Mann-Whitney U test was used to compare the GCF cytokine levels between control group and perio group.

#### Multivariate predictive modeling of chronic periodontitis based on GCF cytokine levels: model selection and validation, and the development of nomograms

Spearman correlations between cytokines were calculated and used as an orientation for model building, in order to prevent redundancies and possible collinearity between cytokines with similar biological effects. Cytokine-based models were selected for their biological significance, their capacity to predict chronic periodontitis and their statistical validity. The biological criteria to select the predictor cytokines are based on their importance level in the inflammatory process, and particularly the different role of pro-inflammatory and anti-inflammatory cytokines.

Models were constructed by initially selecting one pro-inflammatory cytokine as predictor variable. In order to test whether the predictive ability of pro-inflammatory cytokines can be increased by incorporating cytokines with anti-inflammatory effects, two-variable models combining these different mediators were analysed. Resulting models were adjusted individually in relation to the “smoking status” (a non-smoking status was established as the reference).

The statistical criterion applied for model selection was the capacity of each GCF cytokine-based model to discriminate the presence of chronic periodontitis, that was assessed with the Epi package and using the receiver operating characteristic (ROC) curve^35^. This curve was created by plotting the true positive rate (TPR; sensitivity) *versus* the false positive rate (FPR; 1-specificity) at various threshold settings of the analyses. The area under the curve (AUC), which is the C index, was regarded as a measure for the discriminative capacity of the model and provides a scale from 0.5 to 1.0 (with 0.5 representing random chance and 1.0 indicating perfect discrimination) with which to compare the ability of a biomarker to detect a positive result^36^. Note that models with an AUC value equal to or higher than 0.70 are typically considered to be acceptable predictive models^37^. The calculation of the AUC values and their corresponding 95% CIs by bootstrapping were performed using the pROC package^38^. Those models that presented higher AUC values were selected.

Of the selected cytokine-based models, using the pROC package and bootstrapping, numerous classification measures such as accuracy (ACC), sensitivity, specificity, the positive predictive value (PPV), and the negative predictive value (NPV) were obtained by setting an optimal threshold, as well as their corresponding 95% CIs^38^. The best cut-off value for each model was determined so that the percentage of correct predictions was the maximum. As a single indicator of diagnostic performance, the diagnostic odds ratio (DOR) was calculated as the ratio of the odds of positivity in the diseased patients relative to the odds of positivity in those with no disease^39^.

The Hosmer–Lemeshow test was applied to the selected cytokine-based models, using the Resource Selection package^40^. This is a calibration measure, which is significant for poorly calibrated models^41^. Calibration curves of these models were constructed graphically using the rms package^42^ to assess the agreement between the actual outcomes and the predicted probabilities of chronic periodontitis. In a well-calibrated model, the predictions should fall on a 45-degree diagonal line^41^.

The nomograms were built based on the results of multivariable analyses using the rms package^42^. A nomogram maps the predicted probabilities into points on a scale from 0 to 100 in a user-friendly graphical interface. The total points accumulated by the various covariates correspond to the predicted probability of disease for a patient^43^.

#### Internal validation

Bootstrap methods were used to test for possible overfitting by determining optimism values on the discrimination, classification and calibration measures. The bootstrap analysis was replicated on 1000 different samples of the same sample size drawn with replacements from the original sample. Optimism, which is a measurement of internal model validation that refers to the absolute magnitude of bias, equals the difference between respective statistics of the bootstrap sample (bootstrap performance) and the bootstrap model in the original sample (test performance)^44, 45^.

**Figure 2:**
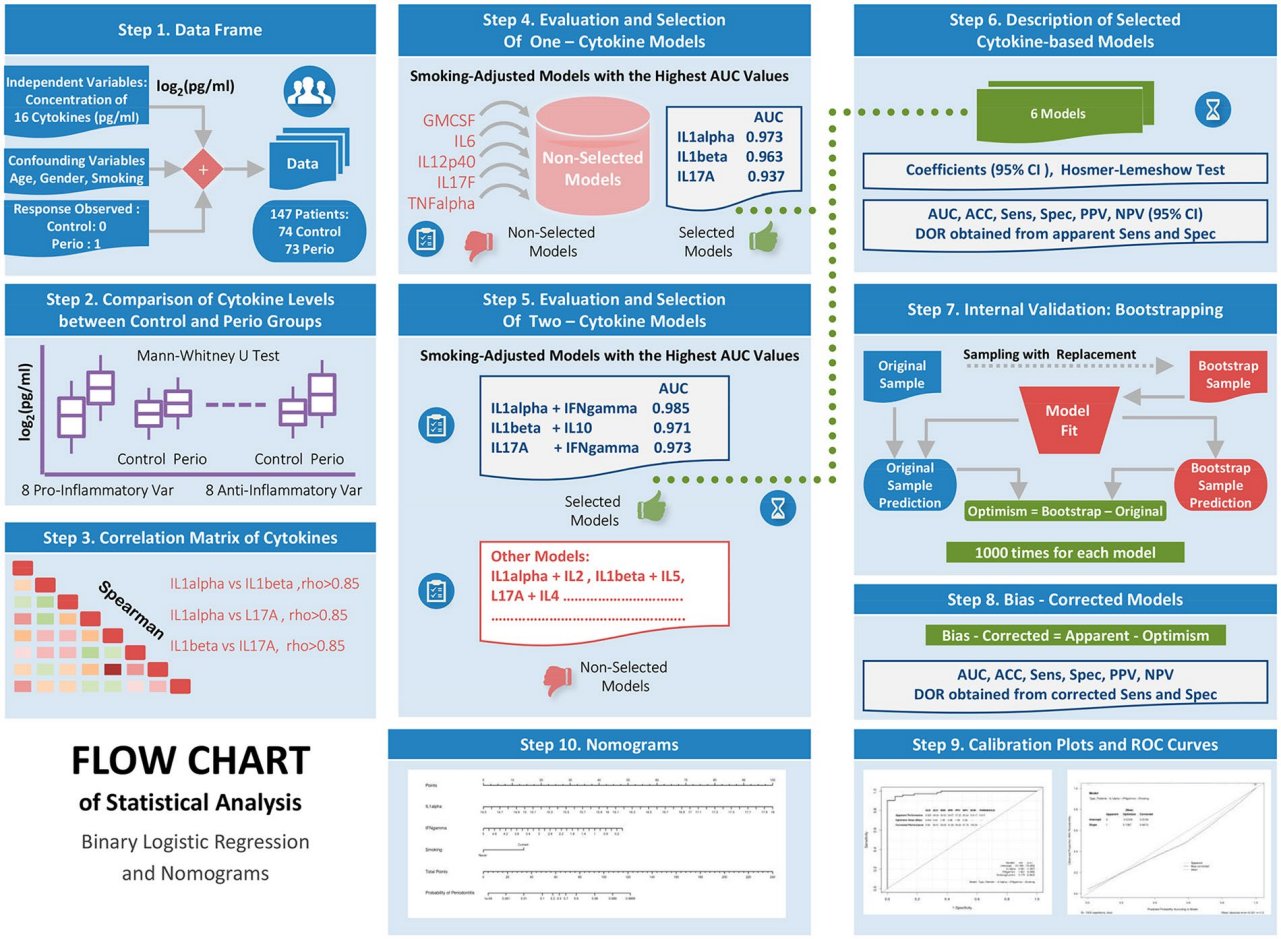
Flow chart of statistical analysis: binary logistic regression and diagnostic nomograms.

Bias-corrected (bc) AUC, all the classification measures (bc-sensitivity, bc-specificity, bc-PPV, bc-NPV), bc-calibration measures were calculated as their corresponding apparent measures derived from the entire original sample minus optimism^44, 45^. In terms of the bc-DOR, these ratios were calculated from the values of bc-sensitivity and bc-specificity. Figure 2 shows the flow chart of statistical analysis: binary logistic regression and diagnostic nomograms.

### Declarations

#### Ethics approval and consent to participate

The study’s protocol was approved by the Clinical Research Ethics Committee of Galicia (number 2015/006). Patients who agreed to participate in the research provided written informed consent.

#### Consent for publication

Not applicable.

#### Availability of data and material

All data generated or analysed during this study are included in this published article (and its supplementary information files).

## Results

### Comparison of clinical characteristics between control individuals and patients with chronic periodontitis

Of the 150 patients, who had fulfilled the inclusion criteria and an adequate periodontal diagnosis, 3 patients were excluded for unexpected events (Fig. 1). Of the 147 patients taking part in the study, who had a mean age of 48.37 ± 11.55 years, 62 were male and 85 female. The mean ages of patients of control and perio groups were 45.65 ± 12.37 and 51.12 ± 10.01 years, respectively (*p* = 0.005). In relation to smoking status, the number of smoker patients was significantly higher in the perio group than in the control group (41 and 13 patients, respectively; *p* < 0.001). The analysis of clinical variables related to oral health status showed that, in comparison with the control group, the perio group had significantly higher values of BPL, BOP, PPD and CAL at both the full mouth and sampling site levels (*p* < 0.001; Table 1).

**Table 1:** Age, gender, smoking habit and clinical characteristics associated with the periodontal status in the control and perio groups.

Clinical parameters	Study groups		
	Control group (n= 74)	Perio group (n= 73)	P Value
**Age (years)**	45.65 (12.37)	51.12 (10.01)	0.005
**Gender**			
Male	31	31	NS
Female	43	42	
**Smoking habit** ^**1**^			
Non-smokers	61	32	<0.001
Smokers	13	41	
Cigarretes/day (no.)	8.08 (4.44)	15.20 (7.94)	0.001
Months of smoking (no.)	236.38 (155.91)	320.78 (109.03)	NS
**No. of teeth**	26.72 (3.25)	25.55 (4.00)	NS
**Full mouth**			
BPL (%)	26.41 (18.66)	53.08 (26.77)	<0.001
BOP (%)	15.05 (6.61)	51.12 (20.07)	<0.001
PPD (mm)	2.11 (0.27)	3.49 (0.65)	<0.001
CAL (mm)	2.36 (0.46)	4.25 (1.12)	<0.001
**Sampled sites**			
BOP (%)	10.11 (10.24)	66.97 (23.93)	<0.001
PPD (mm)	2.23 (0.22)	5.65 (0.89)	<0.001
CAL (mm)	2.31 (0.27)	6.05 (1.12)	<0.001

Values are means (standard deviations) and number of subjects. BPL = bacterial plaque level; BOP = bleeding on probing; PPD = probing pocket depth; CAL = clinical attachment level; NS = not significant. 1-Patients were defined as smokers if he/she was currently smoking and had been a smoker for at least 8 years) and as non-smokers if he/she had never smoked or quitted smoking longer than 5 years before the sampling.

### Comparison of GCF cytokine levels between control individuals and patients with chronic periodontitis

Before the logarithmic transformation, the raw variables were very skewed, which makes difficult to distinguish between individuals with small to moderate cytokine levels. After the logarithmic transformation, the individuals were more regularly spread in the possible values of the transformed variables. Histograms of each cytokine before and after the logarithmic transformation are given in Supplementary Figs S1–16.

The levels of all the pro-inflammatory cytokines (GMCSF, IL1alpha, IL1beta, IL6, IL12p40, IL17A, IL17F and TNFalpha) were significantly higher in the perio group compared to the control group (*p* < 0.001, for all comparisons). Regarding the anti-inflammatory cytokines, only four mediators (IFNgamma, IL2, IL3 and IL4) presented significantly higher concentrations in the perio group (*p* < 0.001, for all comparisons). The increase in the concentrations of these anti-inflammatory cytokines was lower than that detected for the pro-inflammatory cytokines, except for IL3 (Table 2). Boxplots for each cytokine in both study groups are given in Supplementary Figs S17–33.

**Table 2:** Comparison of concentrations of 16 cytokines between both study groups.

Cytokine	Concentration in CGF (log_2_ pg/ml) Median (IQR)		*P* Value
	Control group	Perio group	
GMCSF	7.244 (1.214)	7.954 (1.485)	<0.001
IL1alpha	14.931 (0.889)	17.183 (1.665)	<0.001
IL1beta	11.500 (0.996)	14.132 (1.357)	<0.001
IL6	7.285 (1.956)	8.296 (2.002)	<0.001
IL12p40	3.120 (0.970)	4.173 (0.927)	<0.001
IL17A	3.006 (2.029)	4.867 (1.277)	<0.001
IL17F	2.046 (1.525)	3.373 (1.710)	<0.001
TNFalpha	2.862 (1.697)	4.554 (0.979)	<0.001
IFNgamma	2.417 (1.185)	3.363 (1.395)	0.001
IL2	3.424 (1.133)	3.973 (0.702)	<0.001
IL3	5.607 (1.071)	6.649 (1.880)	<0.001
IL4	2.745 (2.449)	3.713 (2.584)	<0.001
IL5	3.219 (1.301)	3.625 (0.836)	0.495
IL10	2.640 (1.577)	3.101 (2.080)	0.087
IL12p70	3.174 (3.053)	3.864 (1.976)	0.352
IL13	4.904 (2.881)	5.066 (3.514)	0.458

IQR, interquartile range; CGF, crevicular gingival fluid. Concentration range for each biomarker analysed: GMCSF, 0.53–55,050 pg/ml; IFNgamma, 0.02–6,650 pg/ml; IL1alpha, 0.34–28,800 pg/ml; IL1beta, 0.09–23,150 pg/ml; IL2, 0.04–13,700 pg/ml; IL3, 0.19–26,500 pg/ml; IL4, 0.10–29,250 pg/ml; IL5, 0.04–17,800 pg/ml; IL6, 0.10–27,200 pg/ml; IL10, 0.04–10,050 pg/ml; IL12p40, 0.14–27,350 pg/ml; IL12p70, 0.26–18,050 pg/ml; IL13, 0.34–23,700 pg/ml; IL17A, 0.36–30,900 pg/ml; IL17F, 0.25–34,700 pg/ml; TNFalpha, 0.21–16,800 pg/ml.

### Multivariate predictive modeling of chronic periodontitis based on GCF cytokine levels: model selection and validation, and the development of nomograms

For multivariate predictive analysis, we had a total of 147 participants and 73 outcome events. A first description of the relation between cytokine levels is given in Supplementary Dataset 2, by means of their Spearman correlations. Almost all correlations between cytokines, both pro-inflammatory and anti-inflammatory, were positive. The interpretation is that when periodontitis-associated inflammation was present, all cytokines presented larger values. Additionally, we observed larger correlations between pro-inflammatory cytokines. Particularly, strong positive correlations were detected between some very relevant pro-inflammatory cytokines, IL1alpha, IL1beta and IL17A (rho > 0.85). Note also that in the given boxplots these cytokines showed the biggest differences between both study groups.

Respect to the one-cytokine models adjusted by “smoking”, the pro-inflammatory cytokines IL1alpha, IL1beta and IL17 were the predictors that showed higher AUC values (0.973, 0.963, 0.937, respectively). Regarding the two-cytokine models adjusted by “smoking”, the incorporation of certain anti-inflammatory cytokines improved the AUC values of the best models based on a pro-inflammatory cytokine, especially that of IL17A (from 0.937 to 0.974). These two-cytokine models were: IL1alpha + IFNgamma, IL1beta + IL10 and IL17A + IFNgamma. The description of these six models, as well as their corresponding discrimination measures, are detailed in Table 3.

**Table 3:** Description of the six smoking-adjusted models based on cytokine levels, including the apparent and bias-corrected AUC values.

Cytokine-based Models	AUC	bc-AUC
−50.322 + 3.133**IL1alpha** + 1.783	0.973	0.971
−27.729 + 2.136**IL1beta** + 1.722	0.963	0.962
−7.607 + 1.823**IL17A** + 1.860	0.937	0.934
−71.383 + 4.622**IL1alpha** −1.146**IFNgamma** + 2.042	0.986	0.983
−28.811 + 2.331**IL1beta** −0.505**IL10** + 1.701	0.971	0.967
−12.376 + 5.024**IL17A** −3.167**IFNgamma** + 2.984**SmokingCurrent**	0.974	0.971

The IL1beta model presented the highest bc-ACC percentage (93.0%; bc-sensitivity = 92.1%; bc-specificity = 93.9%; bc-DOR = 183.0), followed by the IL1alpha (91.5%; bc-sensitivity = 92.7%; bc-specificity = 90.4%; bc-DOR = 120.7), and IL17A (88.0%; bc-sensitivity = 88.2%; bc-specificity = 87.9%; bc-DOR = 55.0).

In relation to two-cytokine models, the IL1alpha + IFNgamma model and IL1beta + IL10 model showed similar bc-ACC percentages (93.7% and 93.5%; bc-sensitivity = 91.7% and 93.3%; bc-specificity = 95.7% and 93.8%); while the IL17A + IFNgamma model had lower values of bc-ACC (91.2%; bc-sensitivity = 89.1%; bc-specificity = 93.3%). The bc-DOR values for these three models were 249.3, 211.7 and 115.6, respectively. Apparent and bias-corrected classification measures of the six cytokine-based models are described in Table 4. Additional information on these models and their corresponding performance measures are shown in Supplementary Dataset 3.

**Table 4:** Apparent and bias-corrected measures of discrimination and classification of the six smoking-adjusted models based on cytokine levels.

Smoking-adjusted Model	ACC (%)	Sensitivity (%)	Specificity (%)	Positive Predictive Value (%)	Negative Predictive Value (%)	Diagnostic OR
IL1alpha	93.1	94.5	91.8	92.1	94.5	195.5
	91.5	92.7	90.4	90.5	92.9	120.7
Il1beta	93.8	93.1	94.5	94.5	93.4	238.0
	93.0	92.1	93.9	93.8	92.4	183.0
IL17A	89.1	89.0	89.1	89.0	89.2	67.0
	88.0	88.2	87.9	87.8	88.4	55.0
IL1alpha + IFNgamma	95.2	93.1	97.2	97.1	93.5	489.5
	93.7	91.7	95.7	95.6	92.3	249.3
ILbeta + IL10	94.5	94.5	94.5	94.5	94.6	301.9
	93.5	93.3	93.8	93.8	93.4	211.7
IL17A + IFNgamma	92.5	90.4	94.5	94.3	91.0	165.0
	91.2	89.1	93.3	93.1	89.8	115.6

In each cell, the first value is referred to the apparent performance measures and the second, to the corrected performance measures by the level of optimism calculated using a bootstrap procedure. The 95% CIs of the different performance measures, excepting diagnostic OR, are detailed in Supplementary Dataset 3.

**Figure 3:**
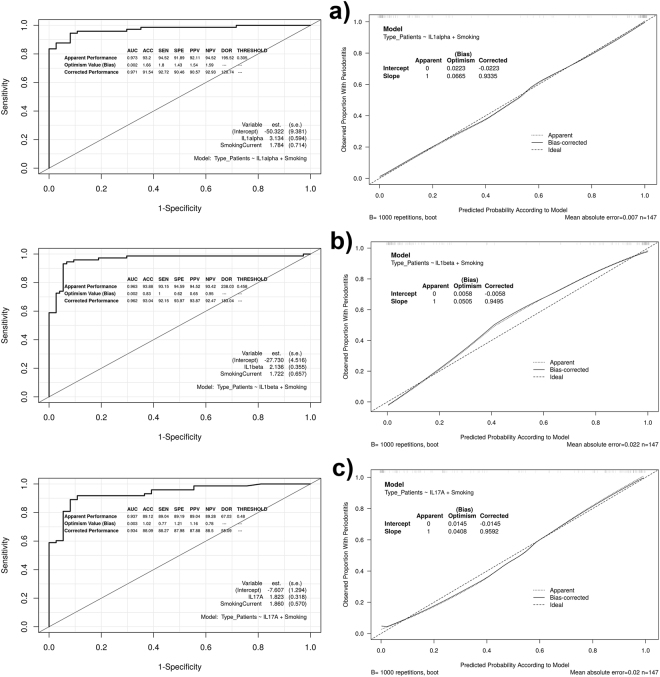
ROC curves and calibration plots of the one-cytokine models, including the apparent and bias-corrected measures by bootstrapping. In the calibration plots, the predicted probability of the model is represented on the x-axis and the observed proportion of chronic periodontitis is represented on the y axis. The 45° line indicates perfect congruity between the predicted probability and the observed proportion of chronic periodontitis.

**Figure 4:**
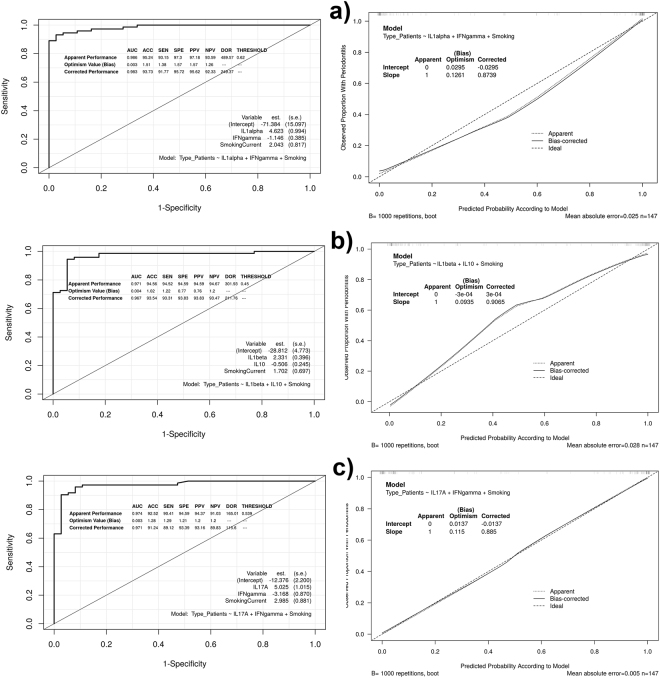
ROC curves and calibration plots of the two-cytokine models, including the apparent and bias-corrected measures by bootstrapping. In the calibration plots, the predicted probability of the model is represented on the x-axis and the observed proportion of chronic periodontitis is represented on the y axis. The 45° line indicates perfect congruity between the predicted probability and the observed proportion of chronic periodontitis.

Figures 3 and 4 show the ROC curves and calibration plots, including the bias-corrected measures, of the six cytokine-based models. The smoking-adjusted models based on IL1alpha and IL17A + IFNgamma were the best calibrated graphically, showing these predictors a linear effect on the outcome. The IL1alpha model presented a bc-intercept of −0.022 and a bc-slope of 0.933, and the IL17A + IFNgamma model, −0.013 and 0.885, respectively. The values of the Hosmer–Lemeshow test were non-significant (*p* = 0.504 and 0.522, respectively).

**Figure 5:**
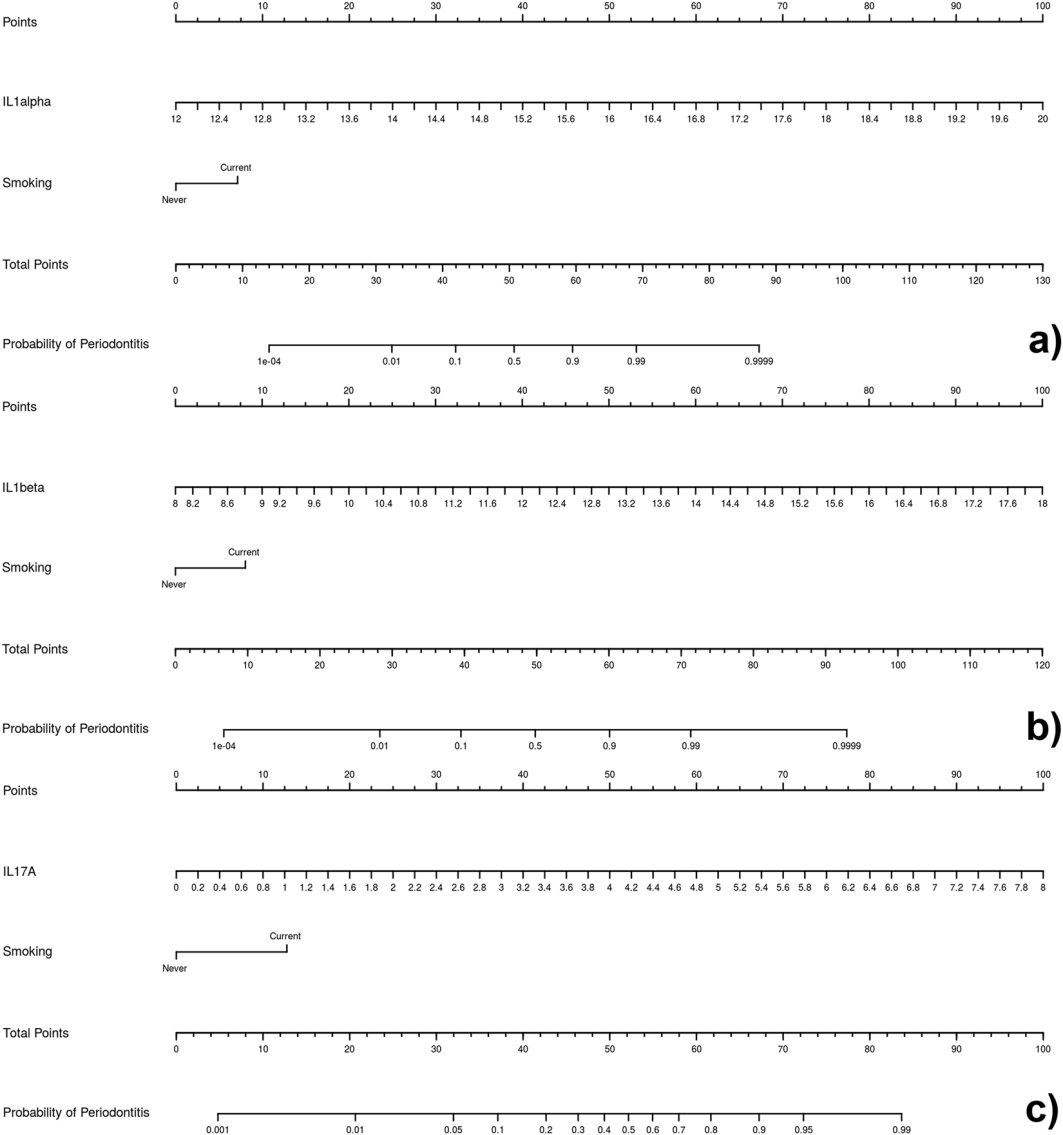
Nomograms based on the one-cytokine models predicting the probability of chronic periodontitis. The probability of chronic periodontitis is calculated by drawing a line to the point on the axis for each of the following variables: (**a**) IL1alpha and “smoking”; (**b**) IL1beta and “smoking”; (**c**) IL17A and “smoking”. The points for each variable are summed and located on the total point line. Next, a vertical line is projected from the total point line to the predicted probability bottom scale to obtain the individual probability of chronic periodontitis.

**Figure 6:**
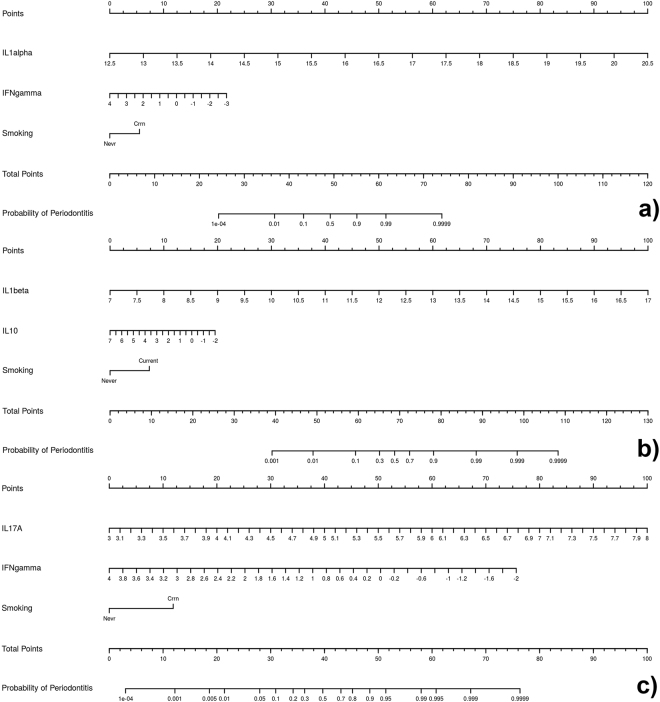
Nomograms based on the two-cytokine models predicting the probability of chronic periodontitis. The probability of chronic periodontitis is calculated by drawing a line to the point on the axis for each of the following variables: (**a**) IL1alpha, IFNgamma and “smoking”; (**b**) IL1beta, IL10 and “smoking”; (**c**) IL17A, IFNgamma and “smoking”. The points for each variable are summed and located on the total point line. Next, a vertical line is projected from the total point line to the predicted probability bottom scale to obtain the individual probability of chronic periodontitis.

Figures 5 and 6 show the diagnostic nomograms derived from the six cytokine-based models. As has been commented upon previously, the discrimination and classification performance values of these nomograms were very high, indicating their outstanding accuracy. According to the calibration parameters, especially the nomogram based on the IL1alpha and IL17 + IFNgamma were very reliable, because these showed very good correspondence between the actual outcomes and the predicted probabilities of having chronic periodontitis. In general, in three nomograms of two cytokines, higher levels of pro-inflammatory cytokines were associated with an increased probability of having chronic periodontitis, as did being a smoker. On the contrary, IFNgamma and IL10 had an opposite function to the pro-inflammatory ones, as higher levels of these mediators were associated with a reduced probability of having periodontitis.

## Discussion

The importance of cytokines in the pathogenesis of periodontal disease has been demonstrated in different stages. Not only do they act as initiators and regulators of the innate and adaptive immune response, but they also mediate the tissue damage that leads to a loss of function and clinical disease^14, 20, 46^. Specifically, cytokines such as GMCSF, IL1beta, IL6, IL17 and TNFalpha are among the more important pro-inflammatory mediators when it comes to stimulating osteoclast activation^17, 18^. On the other hand, anti-inflammatory cytokines such as IL2, IL4, IL5, IL10 and IL13 play a major role in the regulation of T-cell subsets that act at a number of levels, with some of them having an inhibitory effect on osteoclastogenesis (IL13 and IFNgamma)^17, 18^.

### Methodological issues of the published papers

Numerous papers published in the literature have reported the quantification of cytokines in GCF, confirming that there is a distinct cytokine profile for patients with chronic periodontitis^17, 25^. In a recently published meta-analysis study, however, Stadler *et al*.^25^ demonstrated that many of these studies were underpowered (using a small number of subjects and limited assays) and based on a classical reductionist approach because they were focused on just a few cytokines at a time. Although it is generally accepted that the immunopathogenesis of periodontitis is driven by complex and dynamic networks of cytokine interactions^14^, very few authors have evaluated the simultaneous presence of more than 10 cytokines in GCF^26–28^.

In the present study, we used a “knowledge-based” approach and included pro-inflammatory and anti-inflammatory cytokines to construct our panel of 16 biomarkers, selecting those with significant evidence of their role in the pathophysiology of chronic periodontitis. By selecting different classes of cytokines, we aimed to reduce the chances of redundancy among these mediators, potentially improving the diagnostic properties of the multi-cytokine models.

Unlike the position observed in numerous studies^25^, the pooled GCF sample collected from 20 subgingival sites of each patient allowed us to obtain a representative patient-level descriptor of the whole-mouth periodontal inflammatory response at certain times^47^. This can be related to a patient-level periodontitis status and possibly a patient-level disease risk. In the present study, we used a multiplex bead immunoassay for the simultaneous analysis of 16 cytokines in the GCF samples. Despite being a technique applied in less than 20% of the series^25^, these immunoassays provide a sensitivity that is equal to or better than the conventional enzyme-linked immunosorbent assays and have the ability to measure multiple mediators simultaneously (up to 100 different analytes) in volumes as small as 50 µl or less. In contrast, such volumes would only be sufficient for the analysis of a single cytokine using the ELISA technique. Furthermore, the costs per analyte are relatively low and processing times are short^48, 49^. Like the ELISA or any other immunoassay, the sensitivity and specificity of multiplexed microsphere assays depend on the use of high affinity, high specificity antibodies^49^.

The term ‘biomarker’ refers to a biologic compound, which can be quantified and analysed to serve as a biological indicator of physiological or pathogenic processes, environmental exposure and response to therapeutic interventions^50^. Oral fluids such as GCF and saliva, and even blood, have emerged as potential diagnostic tools for the detection of the biomarkers associated with periodontitis^51–53^. However, in terms of the cytokines in GFC, the majority of studies are simply designed to be cross-sectional, in which the cytokine levels in healthy volunteers and periodontal patients are compared for testing, whether or not a particular mediator is increased/decreased at the individual level^20, 25^. There is therefore no evidence in the literature on the development of GCF cytokine-based predictive models for the diagnosis or prognosis of periodontitis using appropriate multivariate predictive modeling techniques^29^. These techniques are useful tools with which to easily evaluate not just a single biomarker, but also a panel of biomarkers, which increases their diagnostic and prognostic power^29^. To the best of the authors’ knowledge, this is the first study in which diagnostic predictive models based on 16 cytokine levels in GCF have been evaluated and validated internally by multivariate predictive modeling techniques.

### Comparison of GCF cytokine levels between control individuals and patients with chronic periodontitis

In order to interpret the large amount of heterogeneous results published in the literature on the GCF cytokine levels in chronic periodontitis, Stadler *et al*.^25^ recently conducted a meta-analysis on the subject. According to this, IL1beta is the most studied cytokine in the pathogenesis of chronic periodontitis, while very few papers have focused on the role of anti-inflammatory cytokines (with IL4 and IL10 being the most researched)^25^. These authors found evidence of significant differences between chronic periodontitis and periodontal health for only a few pro-inflammatory cytokines (IL1beta, IL6 and IL17, which showed higher levels for periodontitis than health). This first conclusion is corroborated by the results of the present study, although we also detected significantly elevated levels of other pro-inflammatory cytokines such as GMCSF, IL1alpha, IL12p40 and TNFalpha. In our series, IL1alpha and IL1beta were the most important pro-inflammatory cytokines in terms of increased concentration associated with the disease, followed by IL6 and GMCSF, and IL17A and TNFalpha.

Equally, in the Stadler meta-analysis^25^, evidence of significant differences between chronic periodontitis and periodontal health was only found for a few anti-inflammatory cytokines (IFNgamma and IL4, which showed higher levels for health than periodontitis). Curiously, however, our results were contrary to those reported by these authors, as four anti-inflammatory cytokines (IFNgamma, IL2, IL3 and IL4) showed significantly higher concentrations in chronic periodontitis than in health. These increases were lower than those detected for the pro-inflammatory cytokines (with the exception of IL3). These discrepancies could be due to methodological differences detected in the studies in relation to the number of subgingival sites sampled and CGF levels obtained, which in many series could be insufficient to determine the levels of anti-inflammatory cytokines.

### Multivariate predictive modeling of GCF cytokine levels: selection of the best models and the development of nomograms

In Periodontology, traditional clinical criteria are often inadequate for: 1) determining sites of active disease; 2) measuring the degree of susceptibility to future progression; or 3) monitoring quantitatively the response to therapy. In this sense, there is a need for research on innovative diagnostic tests based on biomarkers that focus on the early recognition of the microbial challenge to the host, detecting real-time changes in the periodontium^14, 16^. On the other hand, the role of inflammation appears to be the common denominator between periodontitis and some of the systemic diseases and conditions mentioned in the Introduction to this paper. This emphasizes the importance of utilizing oral fluid diagnostic methods for monitoring periodontal diseases^14^. However, to date, limited research has been completed on the use of GCF as a diagnostic measure of periodontal disease^54^.

As an ideal diagnostic test should have predictive accuracy values approaching 100%^17^, in the present series, we surprisingly obtained a number of GCF cytokine-based models (six models) formed by one to two cytokines. These models were supported by the well-known biological role of the cytokines involved, had a capacity to discriminate chronic periodontitis of >0.93 and were statistically validated models. Consequently, they were considered “outstanding predictive models”^37^, and demonstrated that cytokines could be very good biomarkers when it comes to distinguishing patients with chronic periodontitis from periodontally healthy individuals.

There are, however, contrary opinions defending the notion that cytokines are not specific enough for predicting periodontitis, and their levels in the GCF may be affected by local or systemic factors such as smoking, alcohol consumption and stress^15, 55^. It has been stated that cytokine networks are complex, interactive, continuously changing, and have a redundant functionality, meaning that the interpretation of cytokine levels at one particular point in time is fraught with error^56^. In our opinion, however, these affirmations are questionable in the face of the strong evidence on predictive ability of certain cytokines found in the present study.

Regarding the influence of other variables, smoking is a well-established traditional risk factor for chronic periodontitis^57, 58^, and its influence in the levels of some cytokines in the GCF from periodontal patients has been highlighted by previous authors^27, 59^. In the present series, we observed in the GCF cytokine-based models that smoking status increased by 15–20% the probability of having chronic periodontitis.

As there is no literature on the subject, our cytokine-based predictive models could not be compared to similar models, and we have had to consider predictive studies of periodontitis based on other biomarkers detected in the CGF^56, 60^. It is highly unlikely that a single biomarker can be a stand-alone measure for predicting periodontitis activity^15^, and several systemic conditions may affect the GCF levels of a single biomarker^61^.

In the present study, assuming the key role developed by pro-inflammatory cytokines in the pathogenesis of chronic periodontitis^20^, models were constructed by initially selecting one pro-inflammatory cytokine as predictor variable. Surprisingly, the smoking-adjusted models formed by IL1alpha, IL1beta and IL17A showed a high discriminative power (bc-AUC > 0.93), which resulted in corrected percentages of classification measures >87%. Of these pro-inflammatory cytokines, IL1beta was the one that presented the best predictive parameters (corrected classification measures >92%), followed by IL1alpha (corrected classification measures >90%) and IL17A (corrected classification measures >87%). It is very interesting to note the high predictive ability of IL17A, although this cytokine did not show an increase in levels associated with the disease especially high compared to that observed in other pro-inflammatory cytokines. These findings on the outstanding predictive accuracy of these pro-inflammatory cytokines in the CGF has not been previously described in the literature.

However, a combined analysis of different valuable host-responses is required to identify the set of biomarkers with the most favourable combination of sensitivity, specificity and reproducibility^15, 54^. On the other hand, it is advisable to search for smaller multi-biomarker models, which are easier to apply in clinical practice than larger versions^29, 62^. In line with the GCF multi-biomarker predictive models for having peri-implantitis recently described by Zani *et al*.^63^, we analyzed the two-variable models that combined IL1alpha, IL1beta and IL17A with anti-inflammatory cytokines. The purpose was to test if the predictive capacity of these pro-inflammatory cytokines can be increased by the incorporation of cytokines with anti-inflammatory effects. Three smoking-adjusted models were found that fulfilled this premise: IL1alpha + IFNgamma, IL1beta + IL10 and IL17A + IFNgamma (bc-AUC > 0.96; corrected classification measures >89%). To the best of our knowledge, these results are the first evidence on the high predictive ability of models based on a pro-inflammatory cytokine and another anti-inflammatory to distinguish a patient with chronic periodontitis from one with periodontal health.

According to TRIPOD^29^, it was very interesting to test how, although IL10 alone showed non-significant high levels in chronic periodontitis, this acquired an important value within the two-biomarker model, increasing the discriminative capacity of the IL1beta. This corroborates, together with the finding previously commented on IL17A, the importance of designing future studies focused on predictive analysis that are properly powered. In these studies, a large number of periodontitis-related mediators should be evaluated using appropriate multivariate statistical techniques^20, 25^.

In the present series, the best predictive models according to their bc-ACC values were formed by IL1alpha + IFNgamma (bc-AUC > 0.98, bc-ACC > 93% and other corrected classification measures >91%) and IL1beta + IL10 (bc-AUC > 0.96, bc-ACC > 93% and other corrected classification measures >93%), followed by IL17A + IFN gamma (bc-AUC > 0.97, bc-ACC > 91% and other corrected classification measures >89%). This accuracy in terms of predicting chronic periodontitis is much greater than that found in previous studies based on other biomarkers detected in CGF. As a consequence, the model recently validated by Kim *et al*.^60^, which consisted of matrix metalloproteinases (MMP-8, -9, and -13), had an AUC value of 0.84, with a sensitivity and specificity of 70% and 86%, respectively. When other demographic variables and risk factors (age, sex, income, smoking, drinking), as well as blood cytokine levels (IL6, IL8 and TNFalpha), were included in this model, the AUC value increased up to 0.86.

Nomograms are simplified representations of complicated statistical models, and their clinical value relates to the fact that they map the predicted probabilities into points on a scale from zero to 100 in a user-friendly graphical interface^43^. To our knowledge, this is the first study providing several nomograms based on GCF cytokine levels to predict the probability of having chronic periodontitis.

The use of only a few variables is desirable in nomograms to increase their utility in clinical practice^62^. It has been suggested that the proper calibration of a nomogram is more clinically useful than its discriminatory capacity^64^. Our nomograms, which are derived from the one- and two-cytokine models, fulfilled the requirements of discrimination. According to the calibration measures, especially the nomogram based on the IL1alpha and IL17A + IFNgamma were very reliable. If these tools were used in the field of clinical activity, the diagnosis of patients at risk of developing chronic periodontitis could be improved, leading to better, more cost-effective, methods of prevention and treatment of this disease.

On the other hand, Preshaw and Taylor, in an excellent review published in 2011^20^, concluded that cytokines interact and function within networks, but we do not yet understand how the imbalance of the networks relates to the clinical course of periodontitis. In this regard, we are providing evidence through the development of cytokine-based predictive models and their corresponding nomograms. Interestingly, in the nomograms with more than one cytokine, unlike the position with IL1alpha, IL1beta, IL7A and smoking status, higher levels of IFNgamma and IL10 are associated with low scores. This reveals the level of the periodontitis-associated imbalance between these pro-inflammatory (potentiating role) and these anti-inflammatory cytokines (protective role) in terms of a particular probability of having periodontitis.

Our research has some limitations. The most important weakness is that the prediction of the study’s accuracy is only measured in the samples that generated the model equations. As a consequence, to evaluate the reproducibility of the models, we validated the prediction rule internally (calibration, discrimination and classification measures) using bootstrap methods on the original derivation dataset by sampling with replacements for 1000 iterations^45, 64^; the results in this respect were quite optimal. Another limitation was the sample size. Although we are in a scenario of small data, we believe that the concordance between the predictive results obtained and the biological knowledge of the subject indicate that we are on the right way.

The evaluation of these GCF cytokine-based predictive models and nomograms is a potential future research direction. Firstly, it would greatly benefit the strength of our study if the predictive accuracy of the models derived from our series could be measured in an “external” or independent cohort of patients to verify whether our findings are universally applicable. Secondly, the potential prognostic value of these predictive models with regard to clinical progression of the disease and the response to treatment in periodontal patients should be exploited in longitudinal studies, as should their potential predictive accuracy in saliva samples.

In conclusion, of the 16 evaluated cytokines, the GCF levels of the eight pro-inflammatory (GMCSF, IL1alpha, IL1beta, IL6, IL12p40, IL17A, IL17F and TNFalpha) and four anti-inflammatory cytokines (IFNgamma, IL2, IL3 and IL4) were significantly elevated in the patients with chronic periodontitis, with this increase in the concentrations being stronger in the pro-inflammatory cytokines.

The outstanding predictive accuracy of the resulting smoking-adjusted models showed that IL1alpha, IL1beta and IL17A in GCF are very good biomarkers for distinguishing patients with chronic periodontitis from periodontally healthy individuals. The predictive ability of these pro-inflammatory cytokines was increased by incorporating IFNgamma and IL10.

In the nomograms developed herein, higher levels of these pro-inflammatory cytokines and being a smoker increased the probability of having chronic periodontitis (potentiating role), while IFNgamma and IL10 had the opposite function (protective role). The clinical implications of these predictive tools could include improved patient monitoring and the control of disease activity. However, additional evidence is needed to test the external validity of these GCF cytokine-based models for predicting chronic periodontitis and their value for the clinical use of proposed nomograms.

## Electronic supplementary material

Supplementary file

Dataset 1

Dataset 2

Dataset 3
