# Supplementary material for: Cytokine-based Predictive Models to Estimate the Probability of Chronic Periodontitis: Development of Diagnostic Nomograms
Source: Sci Rep. 2017 Sep 14;7:11580. doi: 10.1038/s41598-017-06674-2 (PMC5599565; doi:10.1038/s41598-017-06674-2)
Supplement: Supplementary file 1 — Supplementary file [file 41598_2017_6674_MOESM1_ESM.pdf]

Cytokine-based Predictive Models to Estimate the Probability of Chronic Periodontitis: Development of Diagnostic Nomograms

Authors: Tomás I*[1], Arias-Bujanda N[1], Alonso-Sampedro M[2], Casares-de-Cal MA[3], Sánchez-Sellero C[3], Suárez-Quintanilla D[1], Balsa-Castro C[1]

Supplementary legends

Supplementary Figures S1-S16. Histograms of each cytokine before and after the logarithmic transformation.

Supplementary Figures S17-S33. Boxplots for each cytokine both in the control group and in the perio group.

Supplementary Dataset 1. Dataframe used for comparative and predictive analysis.

Supplementary Dataset 2. Spearman correlations matrix of cytokines.

Supplementary Dataset 3. Description of the six cytokine-based models, as well as their corresponding discrimination and classification measures.

Supplementary Figures S1-S16

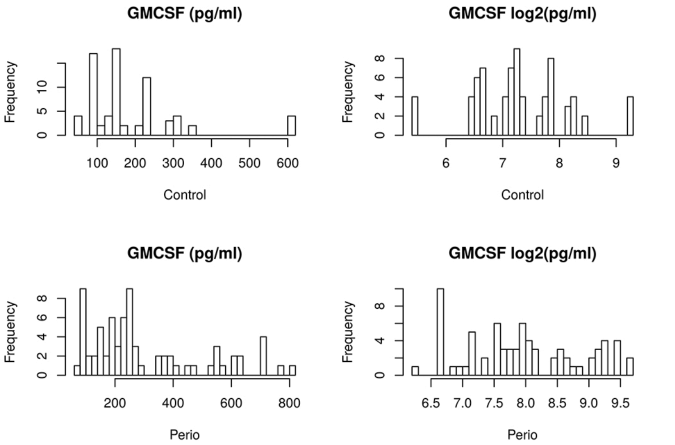
GMCSF (pg/ml)
Frequency
0
5
10
100
200
300
400
500
600
Control
GMCSF log2(pg/ml)
Frequency
0
2
4
6
8
6
7
8
9
Control
GMCSF (pg/ml)
Frequency
0
2
4
6
8
200
400
600
800
Perio
GMCSF log2(pg/ml)
Frequency
0
4
8
6.5
7.0
7.5
8.0
8.5
9.0
9.5
Perio

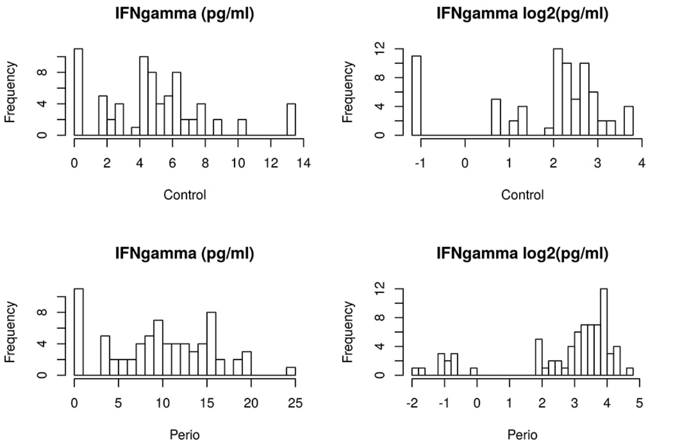
IFNgamma (pg/ml)
Frequency
Control
IFNgamma log2(pg/ml)
Frequency
Control
IFNgamma (pg/ml)
Frequency
Perio
IFNgamma log2(pg/ml)
Frequency
Perio

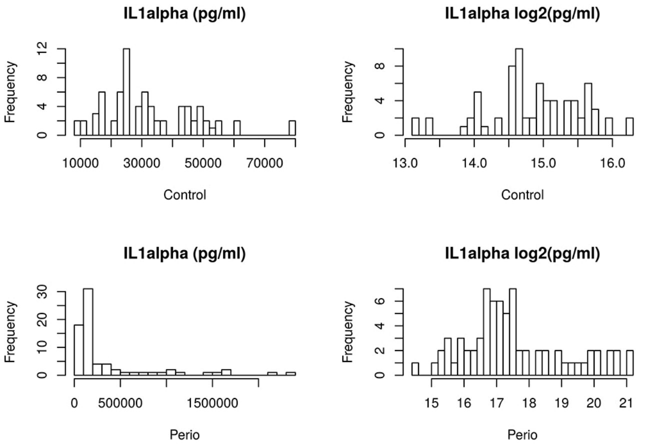
IL1alpha (pg/ml)
Frequency
0
4
8
12
10000
30000
50000
70000
Control
IL1alpha log2(pg/ml)
Frequency
0
4
8
13.0
14.0
15.0
16.0
Control
IL1alpha (pg/ml)
Frequency
0
10
20
30
0
500000
1500000
Perio
IL1alpha log2(pg/ml)
Frequency
0
2
4
6
15
16
17
18
19
20
21
Perio

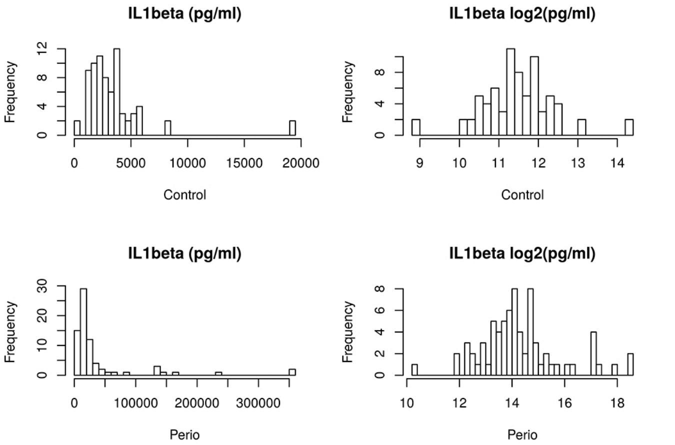
IL1beta (pg/ml)
Frequency
0
4
8
12
0
5000
10000
15000
20000
Control
IL1beta log2(pg/ml)
Frequency
0
4
8
9
10
11
12
13
14
Control
IL1beta (pg/ml)
Frequency
0
10
20
30
0
100000
200000
300000
Perio
IL1beta log2(pg/ml)
Frequency
0
2
4
6
8
10
12
14
16
18
Perio

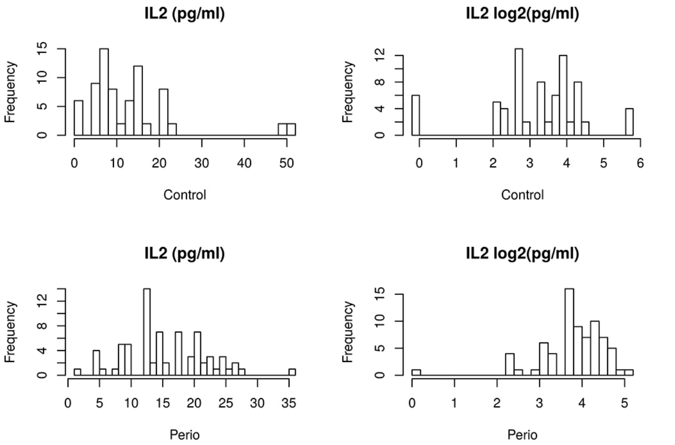
IL2 (pg/ml)
Frequency
Control
IL2 log2(pg/ml)
Frequency
Control
IL2 (pg/ml)
Frequency
Perio
IL2 log2(pg/ml)
Frequency
Perio

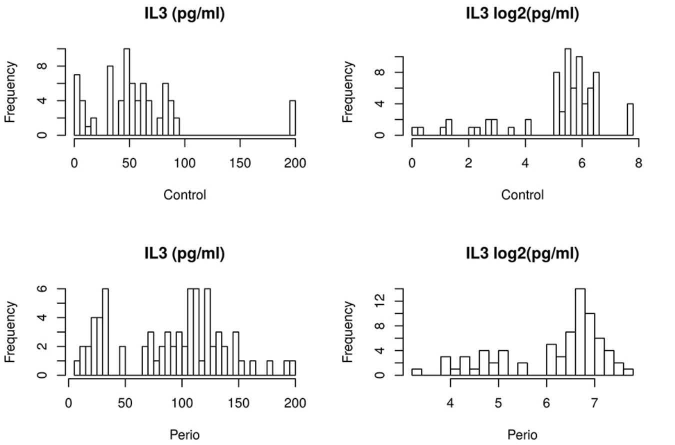
IL3 (pg/ml)
Frequency
Control
IL3 log2(pg/ml)
Frequency
Control
IL3 (pg/ml)
Frequency
Perio
IL3 log2(pg/ml)
Frequency
Perio

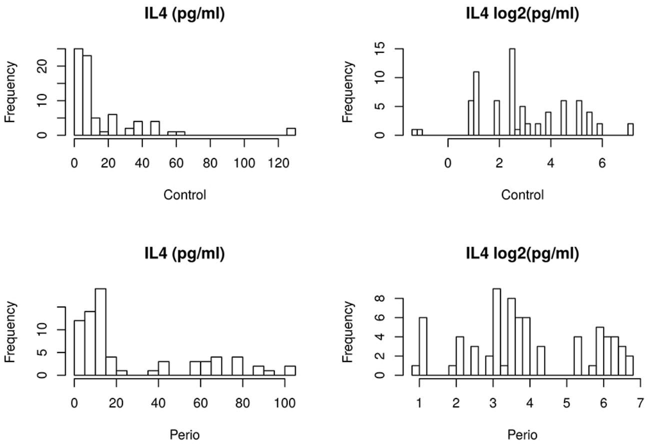
IL4 (pg/ml)
Frequency
Control
IL4 log2(pg/ml)
Frequency
Control
IL4 (pg/ml)
Frequency
Perio
IL4 log2(pg/ml)
Frequency
Perio

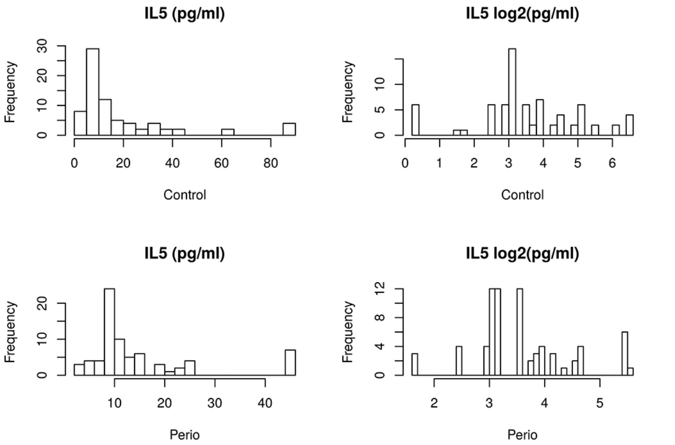

IL5 (pg/ml)
Frequency
Control
IL5 log2(pg/ml)
Frequency
Control
IL5 (pg/ml)
Frequency
Perio
IL5 log2(pg/ml)
Frequency
Perio

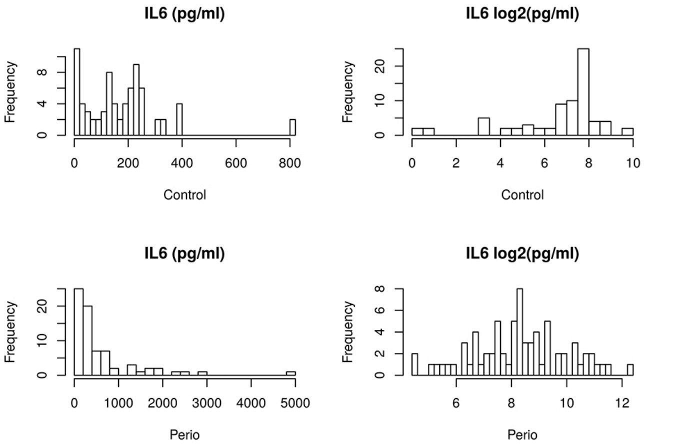
IL6 (pg/ml)
Frequency
0
4
8
0
200
400
600
800
Control
IL6 log2(pg/ml)
Frequency
0
10
20
0
2
4
6
8
10
Control
IL6 (pg/ml)
Frequency
0
10
20
0
1000
2000
3000
4000
5000
Perio
IL6 log2(pg/ml)
Frequency
0
2
4
6
8
6
8
10
12
Perio

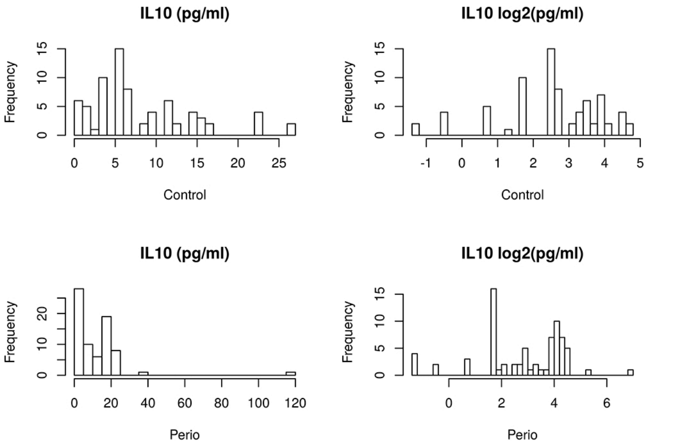

IL10 (pg/ml)
Frequency
Control
IL10 log2(pg/ml)
Frequency
Control
IL10 (pg/ml)
Frequency
Perio
IL10 log2(pg/ml)
Frequency
Perio

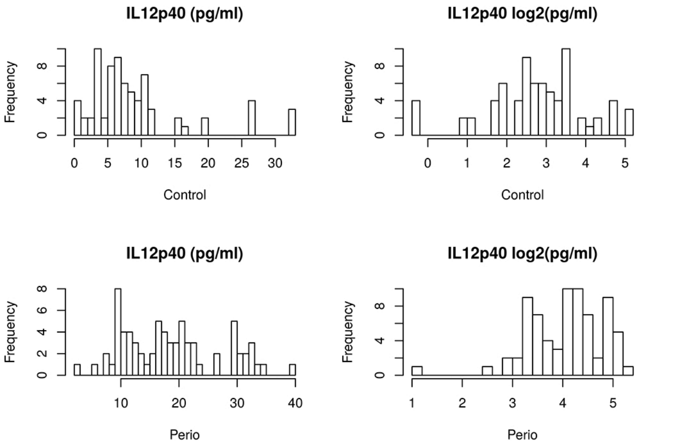
IL12p40 (pg/ml)
Frequency
Control
IL12p40 log2(pg/ml)
Frequency
Control
IL12p40 (pg/ml)
Frequency
Perio
IL12p40 log2(pg/ml)
Frequency
Perio

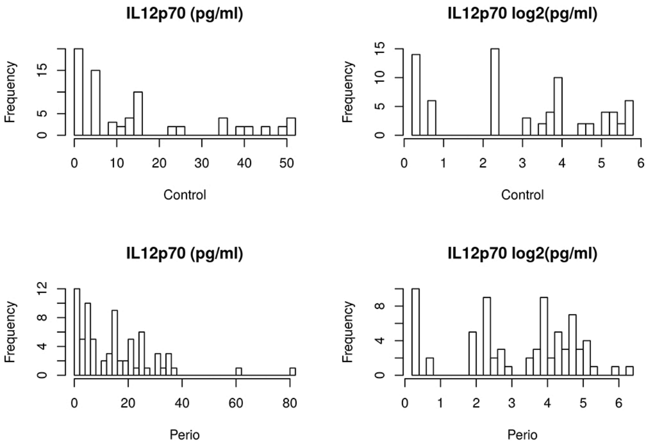
IL12p70 (pg/ml)
Frequency
Control
IL12p70 log2(pg/ml)
Frequency
Control
IL12p70 (pg/ml)
Frequency
Perio
IL12p70 log2(pg/ml)
Frequency
Perio

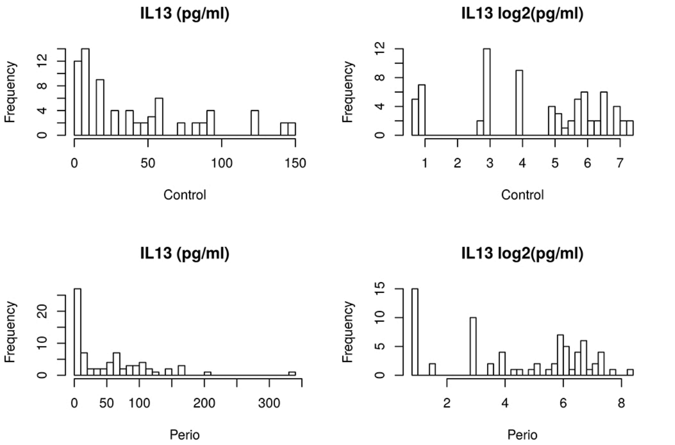

IL13 (pg/ml)
Frequency
0
4
8
12
0
50
100
150
Control
IL13 log2(pg/ml)
Frequency
0
4
8
12
1
2
3
4
5
6
7
Control
IL13 (pg/ml)
Frequency
0
10
20
0
50
100
200
300
Perio
IL13 log2(pg/ml)
Frequency
0
5
10
15
2
4
6
8
Perio

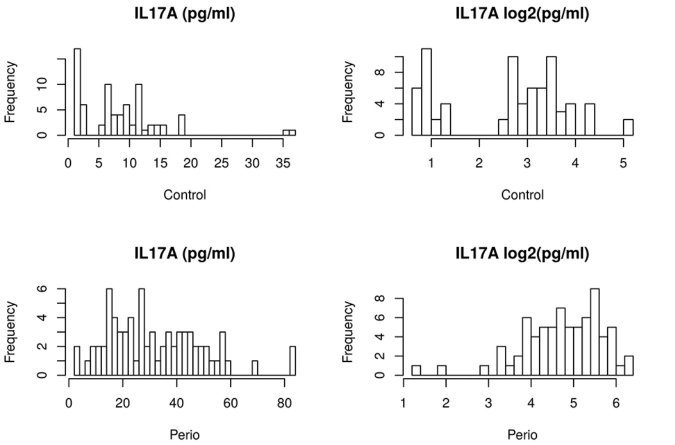

IL17A (pg/ml)
Frequency
0
5
10
0
5
10
15
20
25
30
35
Control
IL17A log2(pg/ml)
Frequency
0
4
8
1
2
3
4
5
Control
IL17A (pg/ml)
Frequency
0
2
4
6
0
20
40
60
80
Perio
IL17A log2(pg/ml)
Frequency
0
2
4
6
8
1
2
3
4
5
6
Perio

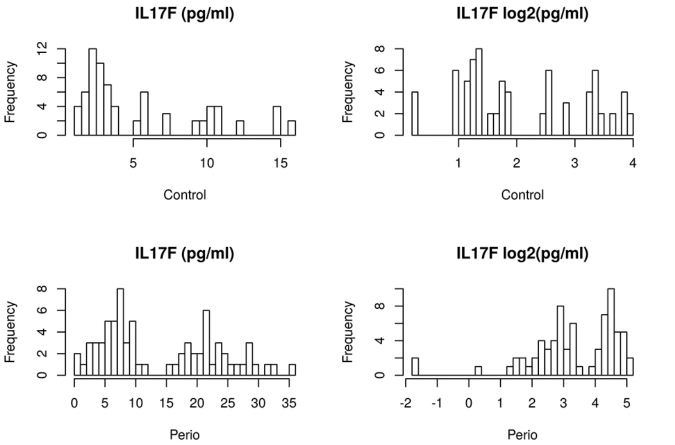
IL17F (pg/ml)
IL17F log2(pg/ml)
Frequency
Control
IL17F (pg/ml)
IL17F log2(pg/ml)
Frequency
Perio

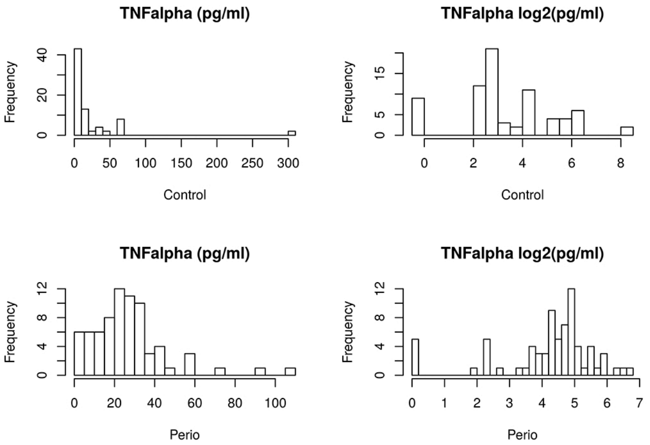

TNFalpha (pg/ml)
Frequency
0
20
40
0
50
100
150
200
250
300
Control
TNFalpha log2(pg/ml)
Frequency
0
5
15
0
2
4
6
8
Control
TNFalpha (pg/ml)
Frequency
0
4
8
12
0
20
40
60
80
100
Perio
TNFalpha log2(pg/ml)
Frequency
0
4
8
12
0
1
2
3
4
5
6
7
Perio

Supplementary Figures S17-S33

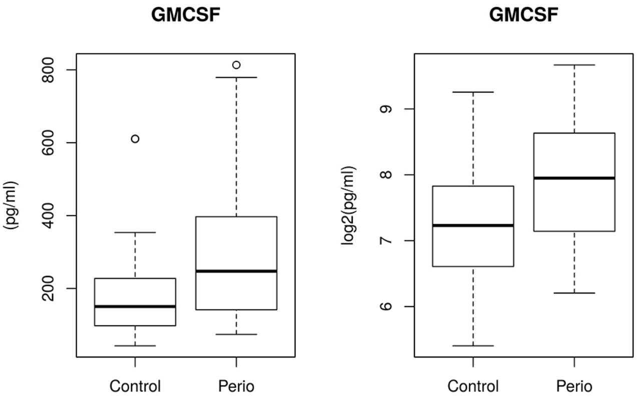
GMCSF
(pg/ml)
800
600
400
200
Control
Perio
GMCSF
log2(pg/ml)
9
8
7
6
Control
Perio

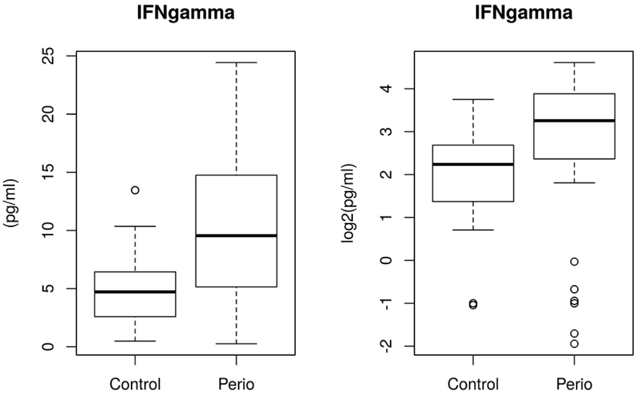
IFNgamma
(pg/ml)
0
5
10
15
20
25
Control
Perio
IFNgamma
log2(pg/ml)
-2
-1
0
1
2
3
4
Control
Perio

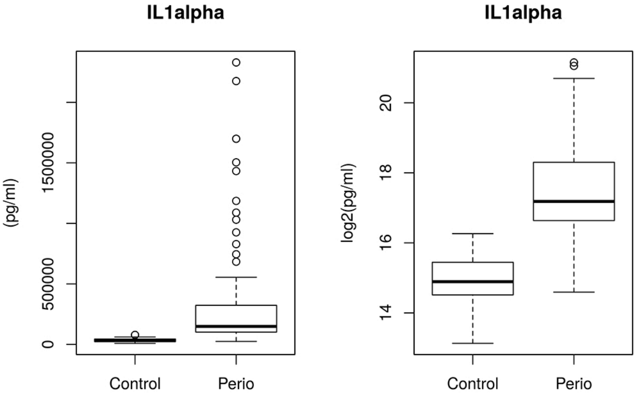
IL1alpha
(pg/ml)
0
500000
1500000
Control
Perio
IL1alpha
log2(pg/ml)
14
16
18
20
Control
Perio

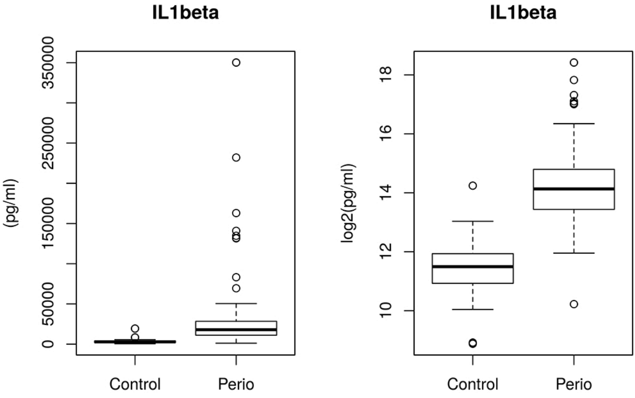
IL1beta
(pg/ml)
0
50000
150000
250000
350000
Control
Perio
IL1beta
log2(pg/ml)
10
12
14
16
18
Control
Perio

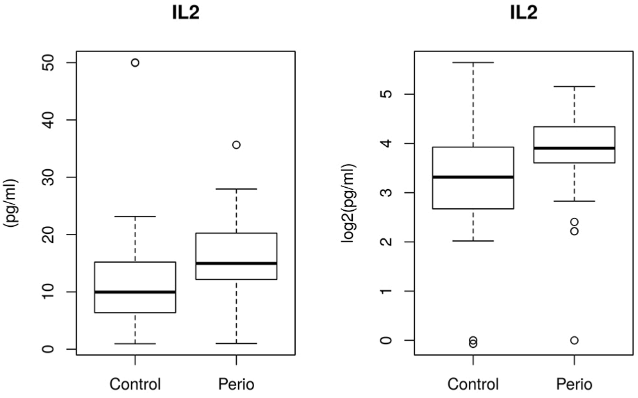
IL2
(pg/ml)
0
10
20
30
40
50
Control
Perio
IL2
log2(pg/ml)
0
1
2
3
4
5
Control
Perio

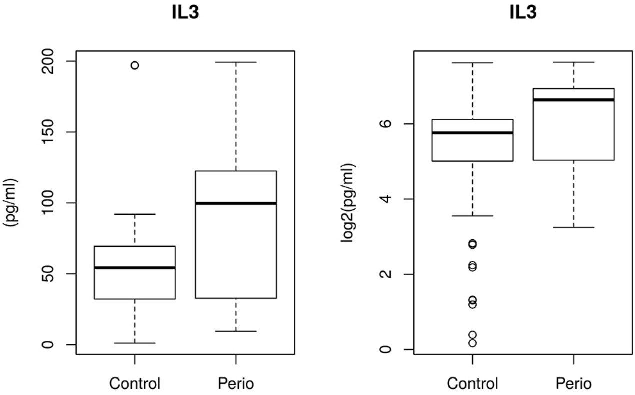
IL3
(pg/ml)
0
50
100
150
200
Control
Perio
IL3
log2(pg/ml)
0
2
4
6
Control
Perio

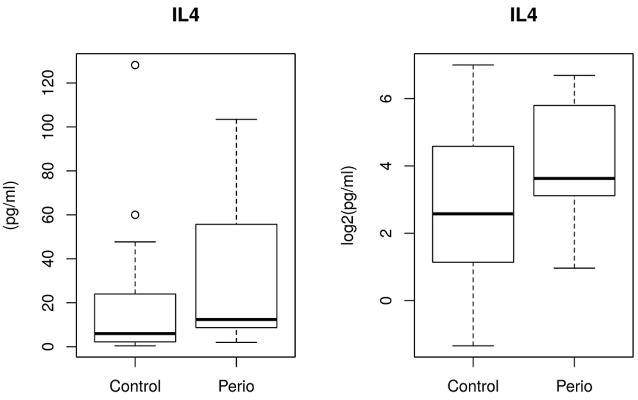
IL4
(pg/ml)
0
20
40
60
80
100
120
Control
Perio
IL4
log2(pg/ml)
0
2
4
6
Control
Perio

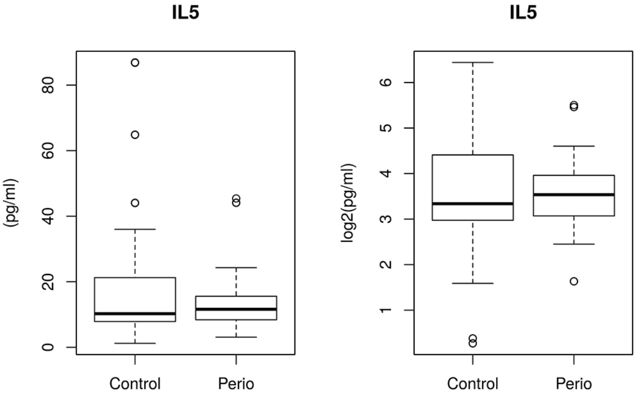
IL5
(pg/ml)
0
20
40
60
80
Control
Perio
IL5
log2(pg/ml)
1
2
3
4
5
6
Control
Perio

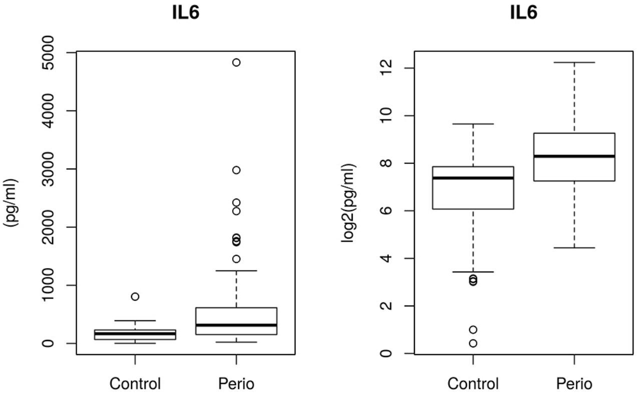
IL6
(pg/ml)
0
1000
2000
3000
4000
5000
Control
Perio
IL6
log2(pg/ml)
0
2
4
6
8
10
12
Control
Perio

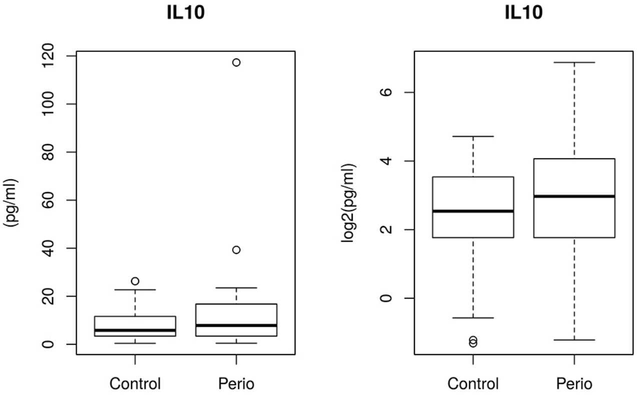
IL10
IL10
(pg/ml)
log2(pg/ml)
0
20
40
60
80
100
120
0
2
4
6
Control
Perio
Control
Perio

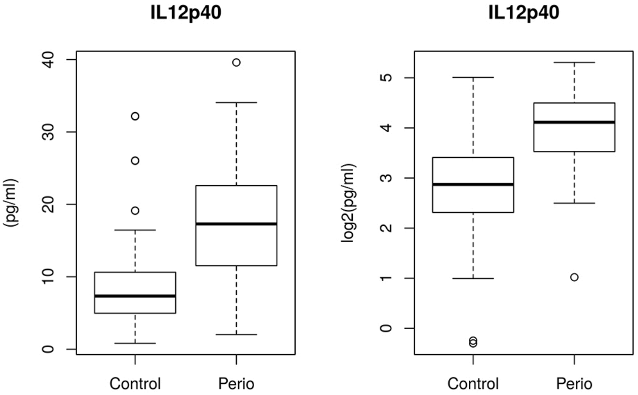
IL12p40
(pg/ml)
0
10
20
30
40
Control
Perio
IL12p40
log2(pg/ml)
0
1
2
3
4
5
Control
Perio

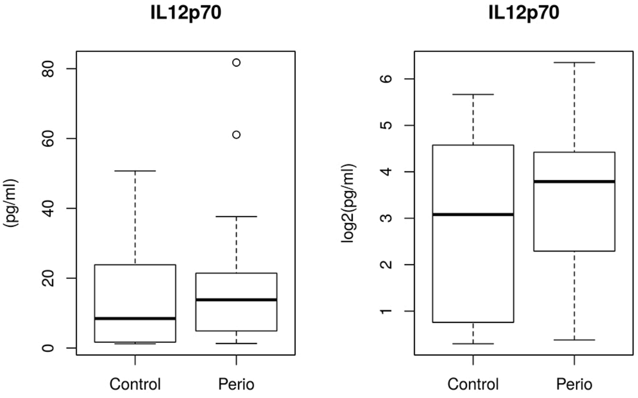
IL12p70
(pg/ml)
0
20
40
60
80
Control
Perio
IL12p70
log2(pg/ml)
1
2
3
4
5
6
Control
Perio

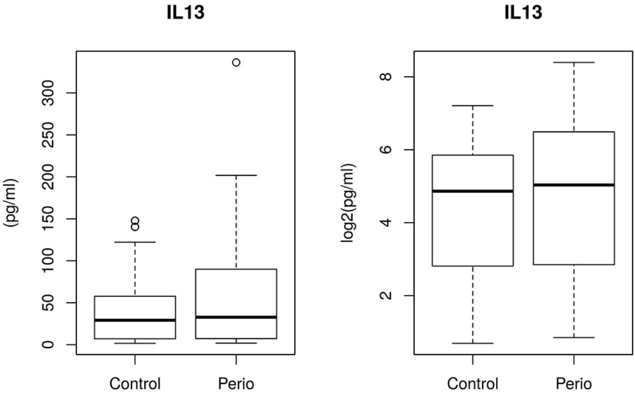
IL13
(pg/ml)
0
50
100
150
200
250
300
Control
Perio
IL13
log2(pg/ml)
2
4
6
8
Control
Perio

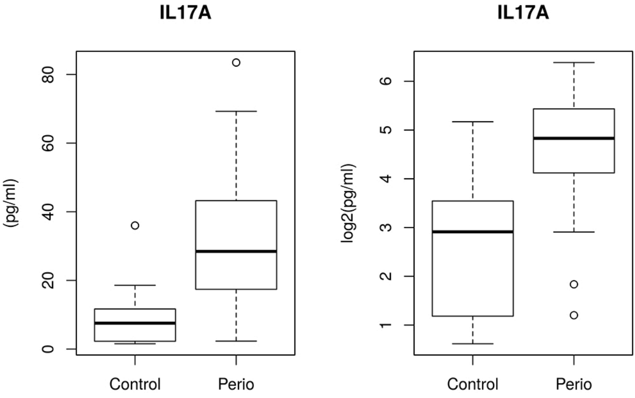
IL17A
(pg/ml)
0
20
40
60
80
Control
Perio
IL17A
log2(pg/ml)
1
2
3
4
5
6
Control
Perio

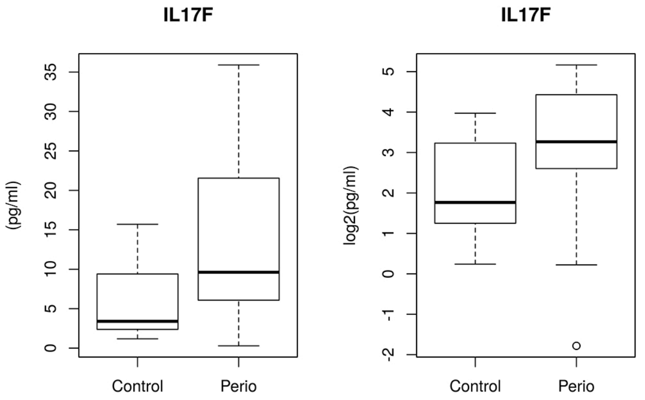

IL17F
(pg/ml)
0
5
10
15
20
25
30
35
Control
Perio
IL17F
log2(pg/ml)
-2
-1
0
1
2
3
4
5
Control
Perio

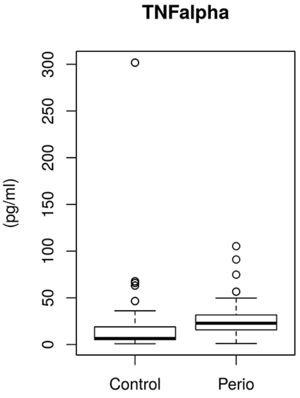

TNFalpha
(pg/ml)
0
50
100
150
200
250
300
Control
Perio

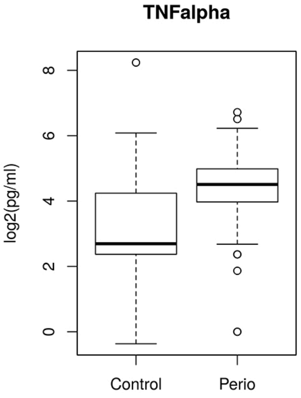

TNFalpha
log2(pg/ml)
0
2
4
6
8
Control
Perio
